# Nonredundant roles of DIAPHs in primary ciliogenesis

**DOI:** 10.1016/j.jbc.2021.100680

**Published:** 2021-04-17

**Authors:** Oliva Palander, Adam Lam, Richard F. Collins, Theo J. Moraes, William S. Trimble

**Affiliations:** 1 Programs in Cell Biology, Hospital for Sick Children, Toronto, Ontario, Canada; 2 Department of Biochemistry, University of Toronto, Toronto, Ontario, Canada; 3 Programs in Translational Medicine, Hospital for Sick Children, Toronto, Ontario, Canada; 4 Department of Paediatrics, University of Toronto, Toronto, Ontario, Canada

**Keywords:** cilia, DIAPH3, DIAPH2, ciliogenesis, cilia maintenance, Actub, acetylated tubulin, Arf4, ADP-ribosylation factor 4, BBSome, Bardet–Biedl syndrome protein complex, CC, coiled coil, Cetn1/CETN1, centrin protein 1, CMV promoter, cytomegalovirus promoter, DAAM, disheveled associated activator of morphogenesis, EB1/ MAPER1, microtubule-associated protein RP/EB family member 1, GFP, green fluorescent protein, Hh, Hedgehog, IFT, intraflagellar transport, INF2 or FHDC1, inverted formin, mDia1/ DIAPH1, mammalian diaphanous protein 1, mDia2/ DIAPH3, mammalian diaphanous protein 3 mDia3/ DIAPH2, mammalian diaphanous protein 2, MT, microtubule, PC-1, polycystin-1, PC-2, polycystin 2, RTKs, receptor tyrosine kinases, TGF- β, transforming growth factor beta, WT, Wild type, Wnt, Wingless

## Abstract

Primary cilia are hubs for several signaling pathways, and disruption in cilia function and formation leads to a range of diseases collectively known as ciliopathies. Both ciliogenesis and cilia maintenance depend on vesicle trafficking along a network of microtubules and actin filaments toward the basal body. The DIAPH (Diaphanous-related) family of formins promote both actin polymerization and microtubule (MT) stability. Recently, we showed that the formin DIAPH1 is involved in ciliogenesis. However, the role of other DIAPH family members in ciliogenesis had not been investigated. Here we show that depletion of either DIAPH2 or DIAPH3 also disrupted ciliogenesis and cilia length. DIAPH3 depletion also reduced trafficking within cilia. To specifically examine the role of DIAPH3 at the base, we used fused full-length DIAPH3 to centrin, which targeted DIAPH3 to the basal body, causing increased trafficking to the ciliary base, an increase in cilia length, and formation of bulbs at the tips of cilia. Additionally, we confirmed that the microtubule-stabilizing properties of DIAPH3 are important for its cilia length functions and trafficking. These results indicate the importance of DIAPH proteins in regulating cilia maintenance. Moreover, defects in ciliogenesis caused by DIAPH depletion could only be rescued by expression of the specific family member depleted, indicating nonredundant roles for these proteins.

Primary cilia function as cellular antennae by receiving signals from various pathways such as Notch, Wingless/wnt (wnt), Hedgehog (Hh), receptor tyrosine kinases (RTKs), and TGF- β (1, 2, 3, 4, 5, 6). Defects in cilia formation or function cause a range of diseases that include skeletal abnormalities, blindness, obesity, cystic kidney, and psychiatric and infertility disorders and are collectively known as ciliopathies (7, 8). In most ciliopathies, cilia possess aberrant morphologies (too long, too short, bulbous tip, etc) (9, 10, 11). To gain insights into the causes of ciliopathies, many studies have focused on the mechanism of ciliogenesis and cilia maintenance.

The initiation of ciliogenesis starts in G1 phase of the cell cycle and cilia continue to grow as cells exit the cell cycle (G0) (12). During the initial phase of ciliogenesis, distal appendage vesicles (DAVs) that most likely derive from the Golgi associate with the distal appendages of the mother centriole (M-centriole). Subsequent steps involve the establishment of the cilia membrane and formation of the transition zone (13, 14, 15), formation of an extracellular cilium membrane channel responsible for fusing the cilium with the plasma membrane (16), and later the development of the ciliary axoneme and cilia membrane. Cilia membrane formation is regulated by Rab family small GTPases, in particular the Rab11-Rab8 cascade (17, 18, 19, 20, 21, 22). The axoneme is formed by polymerization of axonemal microtubules at the growing tip of the projection. Intraflagellar transport (IFT), the bidirectional transit system that carries cargo within the cilium, regulates the elongation process of the distal tip of the axoneme (23, 24, 25, 26, 27, 28). Once cilia have matured, IFTs regulate protein trafficking inside the cilia, while several pathways regulate protein transport to the base of the cilia. Cilia maintenance is regulated by the balance of protein traffic into and within the cilium (29). The trafficking of vesicles to cilia involves IFT20 (30), BBS proteins (17, 31), and exocyst-associated small Rab GTPases (32, 33) and is regulated by a network of microtubules and actin filaments that vesicles use as tracks to reach specific docking sites at the periciliary base (27). In all these pathways, membrane proteins require active transport to cross the ciliary gate and enter the ciliary membrane (29). On the other hand, some membrane proteins such as smoothened have been shown to traffic from the apical membrane into the ciliary membrane *via* lateral diffusion (34). It has been shown that premature exit of membrane proteins from cilia is prevented by the septin-driven formation of a diffusion barrier complex (35, 36, 37). Recently, BBS proteins and IFT have been shown to function together to cycle ciliary signaling proteins such as G protein-coupled receptors (GPCRs), where BBS proteins mediate the exit of ubiquitinated GPCRs (38). In addition, the entry and exit of BBS proteins is regulated by IFTs, as imported BBS proteins are transferred from IFT22 to the IFT trains at the ciliary base (39), and their exit from cilia is regulated by IFT25 (40).

The microtubule and actin networks play a major part in vesicle trafficking to the ciliary base during both ciliogenesis and cilia maintenance (27). The formin group of actin nucleators such as DIAPH1-3, INF1, and FMN1-2 are known to bind microtubules and influence their dynamics, leading to cross talk between the two filament classes (41). To date, DAAM1 and INF1 functions have been linked to motile and primary cilia, respectively (42, 43). More recently, we showed a role for DIAPH1, a member of the Diaphanous formin family, in primary cilia function and ciliogenesis (44). The Diaphanous group of formins, originally discovered in *Drosophila* (45), are a key Rho-regulated family of actin nucleators (46) that also influence microtubule stability (47). The human homologues of *Drosophila* Diaphanous are called DIAPH1, DIAPH2, and DIAPH3 and are orthologs of mouse mDia1, mDia3, and mDia2, respectively (48). DIAPH1-3 are multidomain proteins, where their N-terminal consists of the GTPase-binding domain (GBD). The activation of DIAPH1-3 involves interaction of small GTP-binding proteins of the Rho subfamily to the GBD domain. Rac, Cdc42, and RhoA activate DIAPH2 and DIAPH3, whereas DIAPH1 is only activated by RhoA and RhoC. The GBD domain is flanked by FH1 and FH2, where FH1 is responsible for the interaction with profilin (49), and FH2 is involved in actin polymerization and microtubule stability (50, 51). DIAPH family proteins have been implicated in many cellular processes such as filopodia formation, cell polarization, cell migration, cytokinesis, and chromosome alignment (52). Interestingly, DIAPH2 and DIAPH3 are implicated in vesicle trafficking, as they were shown to govern endosome dynamics (53, 54, 55). With respect to disease, transgenic mice overexpressing wild-type DIAPH3 showed progressive hearing loss with profound abnormalities of inner hair cell (IHC) stereocilia (short, fused, elongated, spare) and loss of IHC ribbons (56, 57). Similar hearing loss has been seen in humans with mutations in a transcriptional regulatory site of DIAPH3 that cause overexpression of DIAPH3 (52, 58). This leads to progressive hearing loss called AUNA1 (auditory neuropathy, nonsyndromic, autosomal dominant, 1). On the other hand, a mutation in DIAPH2 in humans is linked to premature ovarian failure (POF) (59, 60), and other defects in ciliogenesis have been linked to POF (61). Our previous work revealed a role for DIAPH1 in ciliogenesis and cilia maintenance (44), and these studies raise the untested possibility that DIAPH2 and/or DIAPH3 may also be important in the formation of primary cilia.

Here, we investigated the roles of DIAPH2 and DIAPH3 in ciliogenesis and cilia maintenance. Using siRNA-mediated depletion of DIAPH2 and DIAPH3, we observed impaired ciliogenesis, decreases in cilia length, and decreases in IFT20 or Rab11 recruitment to the ciliary base. By specifically targeting DIAPH3 to the basal body of cilia, we observed elongation of cilia and the formation of bulbous cilia tips. Moreover, targeting DIAPH3 increased trafficking of post-Golgi vesicle (IFT20) and early/recycling endosomes to the base of cilia. Our data suggests a model in which DIAPH2 and DIAPH3 proteins contribute nonredundantly to ciliogenesis and suggests that DIAPH3 may coordinate vesicle trafficking to the base of cilia to regulate ciliogenesis and cilia maintenance.

## Results

### DIAPH2 and DIAPH3 function in ciliogenesis and localize to the ciliary base

**Figure 1:**
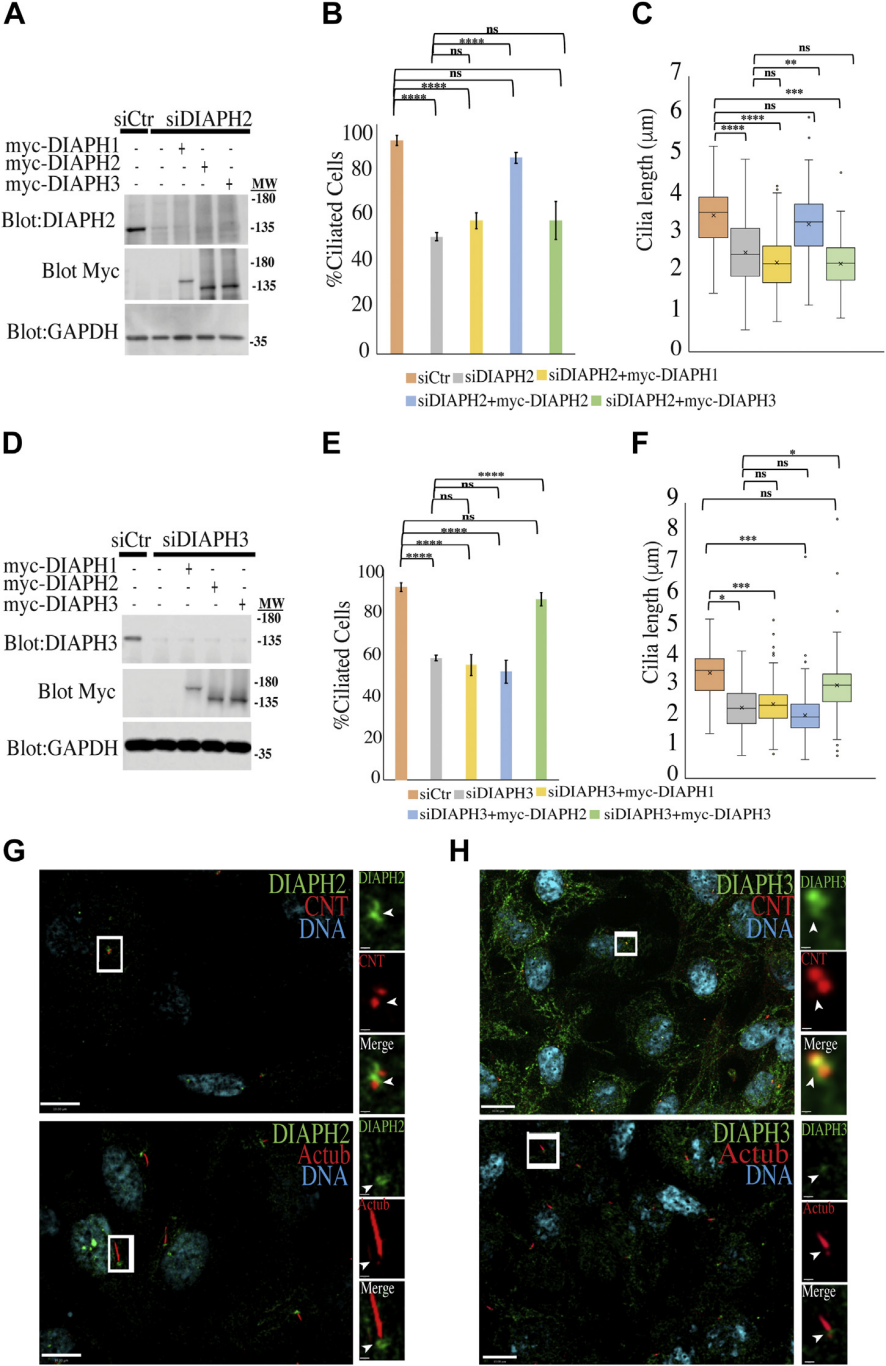
**DIAPH3 regulates ciliogenesis and cilia length.***A–C*, impairment of ciliation and cilia length by DIAPH2 depletion cannot be rescued by other isoforms. hTERT-RPE1 cells were transfected with siCtr or siDIAPH2#1 for 48 h, and cells were rescued using wild-type constructs of DIAPH1-myc, DIAPH2-myc, and DIAPH3-myc. *A*, western blots of samples blotted for human DIAPH2, myc, and GAPDH. Quantifications of (*B*) ciliation and (*C*) cilia length are shown. Error bars in B represent ± SD of three independent experiments; n = 50 or more each. Graph of cilia length measurement (*C*) is displayed in box-and-whisker plot, where the upper and lower quartiles are indicated as the ends of the box, the median is marked by a horizontal line inside the box, mean is marked by a cross sign inside the box, and whiskers are two lines outside the box that extend to the highest and lowest observations, while dots outside this range represent outliers. Two-tailed *t*-test analysis was done to compare control siRNA to all samples or siDIAPH1 to rescue samples, ∗*p* < 0.05, ∗∗*p* < 0.01, ∗∗∗*p* < 0.005, ∗∗∗∗*p* < 0.001. *D–F*, reduction of ciliation and cilia length by DIAPH3 depletion cannot be rescued by other isoforms. hTERT-RPE1 cells were transfected with siCtr or siDIAPH3#1, and cells were rescued using wild-type constructs of DIAPH1-myc, DIAPH2-myc, or DIAPH3-myc. *D*, western blots of rescued cells blotted for human DIAPH3, myc, and GAPDH. *E* and *F*, quantification of ciliation and cilia length in hTERT-RPE1 cells treated with siRNA for 48 h, respectively. Error bars in E represent ±SD of three independent experiments; n = 50 or more each. Two-tailed *t*-test analysis was done to compare control siRNA to all samples or siDIAPH1 to rescue samples, ∗*p* < 0.05, ∗∗*p* < 0.01, ∗∗∗*p* < 0.005, ∗∗∗∗*p* < 0.001. Graph of cilia length measurement is displayed in a box-and-whisker plot as in C. *G* and *H*, DIAPH2 and DIAPH3 localize at the base of cilia. hTERT-RPE1 cells were grown to confluence and serum starved for 24 h. Cells were stained with Actub (axonemal marker) and centrin (CNT) for cilia basal marker. Cells were costained for (*G*) DIAPH2 and CNT or DIAPH2 and Actub. *H*, cells were costained for DIAPH3 and CNT or DIAPH3 and Actub. Scale bars in G and H are 10 μm in the *left panels* and 1 μm in the *right-side panels*.

Since mutations in DIAPH1-3 gave clinical phenotypes similar to known ciliopathies (62, 63, 64, 65), we have investigated whether DIAPH proteins contribute to ciliogenesis. Recently we showed that DIAPH1 plays a role in ciliogenesis and cilia maintenance (44), so here we examined whether DIAPH2 and DIAPH3 may also play roles in these processes. To do so we used siRNA to individually knock down DIAPH2 or DIAPH3 (Figs. 1, *A*–*F*; S1*A–C*). To control for off-target effects, DIAPH2 and DIAPH3 were each depleted by using two different short interfering RNA (siRNA) sequences (siRNA#1, Fig. 1, *A*–*F* and siRNA#2 Fig. S1, *A–C*) in hTERT-RPE1 cells. Depletion of either DIAPH2 or DIAPH3 caused a reduction in ciliation and cilia length (Fig. 1, B, C, E, F; Fig S1, *B* and *C*) similar to what we had seen previously for DIAPH1. Since cells were serum starved for 24 h upon depletion of DIAPH2 or DIAPH3, we presumed that the effects of DIAPH2 and DIAPH3 depletion on ciliogenesis and cilia length were not due to cell cycle changes. However, to confirm that this is the case, we performed western blotting of lysates of depleted cells with antibodies specific for phosphorylated S807 of the retinoblastoma protein Rb. As shown in Fig. S1*E*, phosphorylated S807 Rb was not detected in the samples treated with siCtr or siDIAPH2 or siDIAPH3, and it was only seen in untransfected cells in the presence of serum (Fig. S1*E*). The specificity of the knockdowns was confirmed by western blotting for each protein individually (Fig. S1, *A* and *I*) and as well by fixing and staining cells for DIAPH2 or DIAPH3 upon their depletion (Fig. S1*D*). To confirm the specificity of the knockdown results and to examine possible redundancy of functions among DIAPH isoforms, myc-tagged mouse homologs of the DIAPH proteins were expressed in depleted cells to rescue the phenotypes. The expression of the murine proteins, which are not detected by human-specific anti-DIAPH antibodies, was shown by blotting for myc (Figs. 1, *A* and *D*, S1*F*). Surprisingly, ciliogenesis and cilia length were rescued in an isoform-specific manner (Fig. 1, *A*–*F*, Fig. S1, *F–H*), suggesting that their functions in ciliogenesis are nonredundant.

To further confirm this nonredundancy of function for DIAPH1-3 in ciliogenesis, we used siRNA to knock down each protein individually or in combination (Fig. S1, *I–K*). Consistent with the idea that each protein is carrying out distinct functions within the same pathway, codepletion of DIAPH1-3 proteins did not result in additive effects. Thus, each of the three DIAPH proteins appears to have a nonredundant, but likely related, role in ciliogenesis.

We previously observed DIAPH1 localization to the ciliary base, so we examined the localization of DIAPH2 and DIAPH3 in ciliated hTERT-RPE1 cells and compared them to acetylated tubulin and centrin to detect the axoneme and basal body, respectively. Using confocal microscopy, we found that DIAPH2 and DIAPH3 also localized with centrin at the base of cilia (Figs. 1, *G* and *H*; S2*A*). We also confirmed this localization in HFF1 cells (human foreskin fibroblast). Additionally, DIAPH1-3 proteins localized to the base of motile cilia in primary human cells from the nasal epithelium (Fig. S2*B*). Since our previous data showed that DIAPH1 localization at the base was regulated by Rho signaling (44), we investigated if Rho signaling played role in the recruitment of DIAPH2 or DIAPH3. Cells were treated with Rho inhibitor that inhibits RhoA, B, and C for either 2 h or 4 h. In contrast to DIAPH1, we found that inhibition of Rho signaling has no effect on recruitment of DIAPH2 or DIAPH3 (Fig. S2, *C–E*). Since all three DIAPHs are important for ciliogenesis and no redundancy in their function was detected, we examined their recruitment during ciliogenesis by measuring their intensity at various time points after serum starvation. Since centrin is located at the basal body and is present throughout ciliogenesis (14, 16), we used it as marker to track ciliogenesis. No change in DIAPH1 levels was detected throughout ciliogenesis, while DIAPH2 increased in intensity at 24 h post starvation and DIAPH3 seemed to decrease at serum starvation, then remained constant throughout ciliogenesis (Fig. S2, *E* and *F*). Altogether, these results indicate that DIAPH isoforms regulate ciliogenesis and cilia length, and they localized in the vicinity of the basal bodies in airway motile cilia and in primary cilia of various cell types.

### DIAPH2 and DIAPH3 regulate recruitment of ciliary proteins

**Figure 2:**
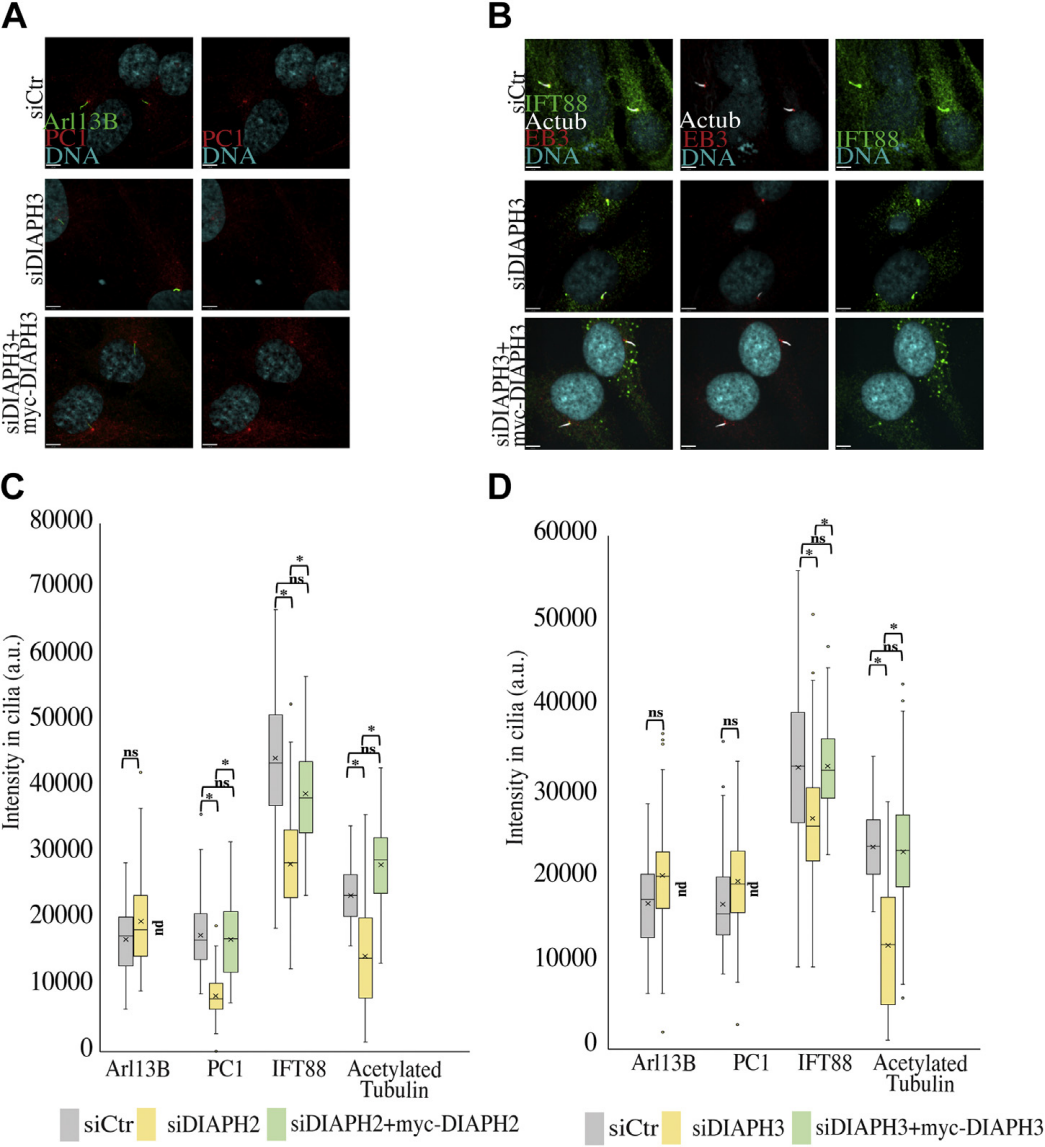
**Decreased trafficking to primary cilia upon depletion of DIAPH2 and DIAPH3.***A–D*, depletion of DIAPH2 or DIAPH3 decreased trafficking to cilia. *A* and *B*, hTERT-RPE1 cells were transfected with siCtr, siDIAPH3, or siDIAPH3 plus the rescue construct myc-DIAPH3 for 48 h. Later cells were fixed and stained for (*A*) Arl13B and PC1 or (*B*) acetylated tubulin, Eb3 (as basal body marker) and IFT88. Scale bars are 5.00 μm for the represented images. *C*, depletion of DIAPH2 reduced in the level of IFT88, PC1, and tubulin acetylation with no change in Arl13B in primary cilia. Box-and-whisker plots (as defined for Fig. 1*C*) are used to display average fluorescence intensity of Arl13B, PC1, acetylated tubulin, and IFT88 upon DIAPH2 depletion and rescue. Fluorescence intensity was measured as the average intensity over the entire cilium (see Fig. S3*E*) and therefore normalized for length. The rescue for Arl13B was not done (nd). *D*, reduction of IFT88 and tubulin acetylation levels was detected upon depletion of DIAPH3. Average fluorescence intensity was measured to determine protein levels of Arl13B, PC1, acetylated tubulin, and IFT88 upon DIAPH3 depletion and rescue. The rescue for Arl13B and PC1 was not done (nd). Box-and-whisker plots are used to display average fluorescence intensity. Three independent experiments; n = 50 cells each. Two-tailed *t*-test analysis was done to compare control siRNA to all protein depleted samples and as well compare siDIAPH2 or siDIAPH3 to rescue samples, ∗*p* < 0.05, ∗∗*p* < 0.01, ∗∗∗*p* < 0.005, ∗∗∗∗*p* < 0.001.

Since we previously showed that depletion of DIAPH1 altered trafficking to cilia (44), we examined whether depletion of DIAPH2 or DIAPH3 also had these effects. Upon singly depleting DIAPH2 or DIAPH3, there was a decrease in the level of acetylated tubulin and a reduction of the intraflagellar trains at the distal tip of cilia (as detected with IFT88) (Fig. 2, *B–D*). In contrast, no change in the level of Arl13B (a ciliary membrane protein involved in membrane protein import) was detected following DIAPH2 or DIAPH3 depletion, while the PC1 membrane protein level was decreased upon depletion of DIAPH2 but not DIAPH3 (Fig. 2, *A*, *C* and *D*). Hence, the surprising observation was that, even though the DIAPH proteins function nonredundantly, they appear to have some common effects on cilia development.

**Figure 3:**
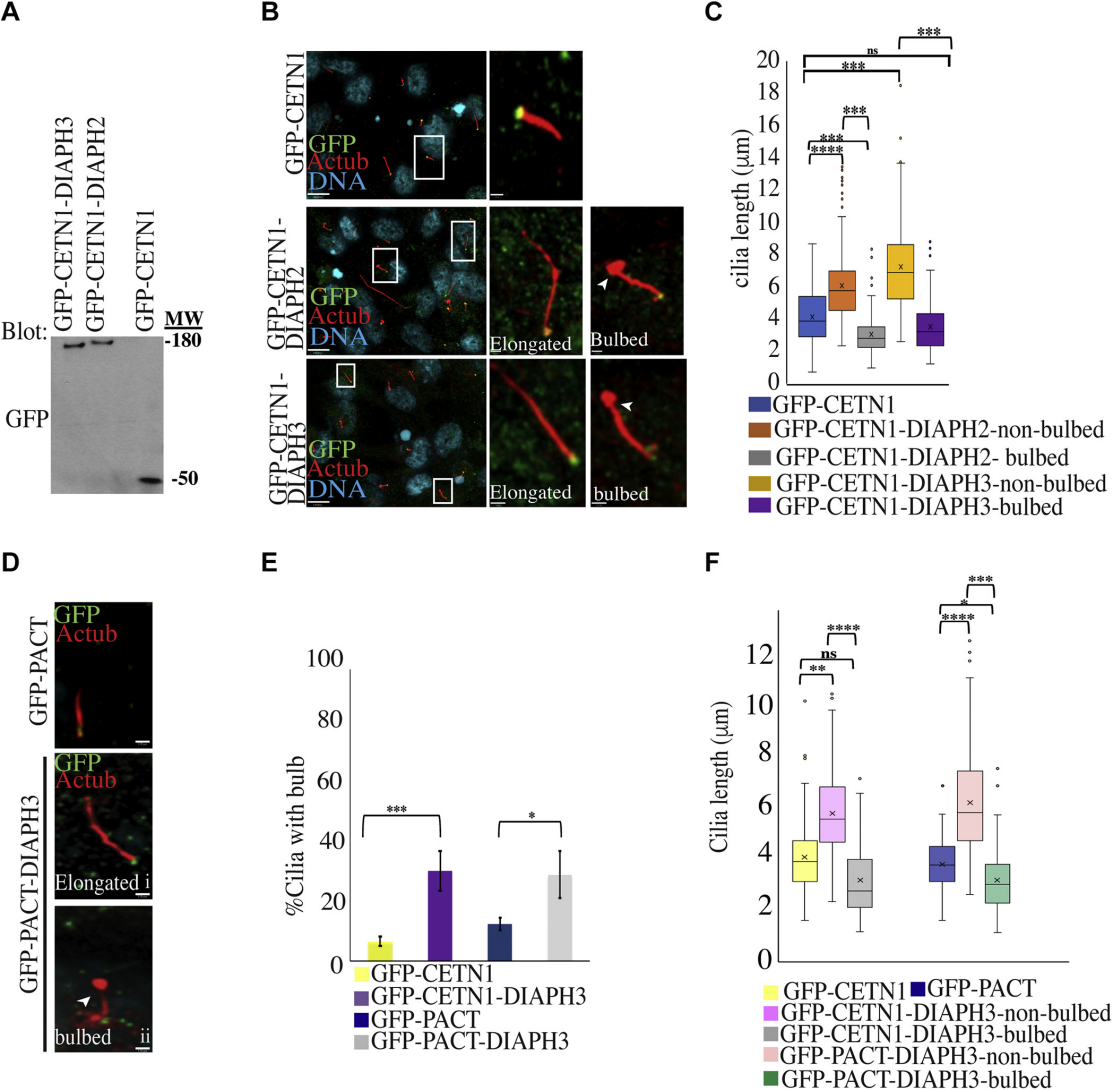
**Targeting DIAPH3 to the ciliary base regulates cilia length and morphology.***A–F*, targeting DIAPH2 and DIAPH3 to the ciliary base *via* centrin or PACT causes elongation and bulb formation at the tip of the cilia. *A*, fusion constructs were made between GFP-centrin (CNTN1) and the indicated proteins (DIAPH2 and DIAPH3). Cells were transfected with GFP-CNTN1 (control), GFP-CNTN1-DIAPH2, or GFP-CNTN1-DIAPH3 and blotted for GFP to detect protein size and to ensure integrity of the fusion proteins. *B*, constructs were transfected into hTERT-RPE1 cells, which were then serum starved for 24 h to induce ciliogenesis, fixed, and stained for acetylated tubulin (cilia marker) and GFP (to detect transfected cells). Cilia are shown in boxes (*left panels*) and the boxed areas are magnified (*right panels*). Scale bars are 8 μm in the left panel and 1 μm in the right-side panels. *Arrowheads* point toward the bulbs at tips of cilia. *C*, quantification of bulbed versus nonbulbed cilia. Measurement of cilia length for GFP-CNTN1, GFP-CNTN1-DIAPH2, or GFP-CNTN1-DIAPH3 samples was presented in Box–Whisker graphs (as in Fig. 1*C*). *D*, targeting DIAPH3 to the ciliary base *via* PACT causes elongation and formation of bulbs at the tip of the cilia. Fusion constructs were made between GFP-PACT (centrosomal targeting domain) and DIAPH3. hTERT-RPE1 cells were transfected with GFP-PACT or GFP-PACT-DIAPH3 and serum starved for 24 h and fixed and stained for GFP and acetylated tubulin. Scale bars are 1 μm for each image. *E–F*, hTERT-RPE1 cells were transfected with CNTN1-GFP (control), GFP-CNTN1-DIAPH1, GFP-CNTN1-DIAPH3, GFP-PACT (control), GFP-PACT-DIAPH1, or GFP-PACT-DIAPH3. Cells were serum starved for 24 h to induce ciliogenesis. Quantification for ciliated cells with bulb (*E*) and length of bulbed versus nonbulbed cilia (*F*) was conducted. Error bars in E represent ± SD of three independent experiments; n = 50 cells each, ∗*p* < 0.05, ∗∗*p* < 0.01, ∗∗∗*p* < 0.005, ∗∗∗∗*p* < 0.001. Two-tailed *t*-test analysis was done to compare CNTN1-GFP control to the samples with GFP-CNTN1, while GFP-PACT control was compared with samples with GFP-PACT. Box and whisker plots (*F*) were performed as in Fig. 1*C*).

To complement the depletion studies, we examined the effect of DIAPH activation or overexpression on cilia growth. Since both DIAPH2 and DIAPH3 were observed at the base of cilia, we examined the effects of targeting them directly to the basal body to prevent global overexpression. We fused full-length DIAPH2 or DIAPH3 to centrin, a basal body protein, which allowed localized increases of DIAPH2 or DIAPH3 at the basal body without affecting DIAPH2 or DIAPH3 levels elsewhere in the cell. hTERT-RPE1 cells were transfected with GFP-centrin (GFP-CETN1) tagged constructs of each protein, and the cells were serum starved to induce ciliogenesis, immunostained, and the length of the cilia was measured. Integrity of the fusion proteins was confirmed by western blotting following immunoprecipitation with anti-GFP antibodies (Fig. 3*A*). Using confocal microscopy to detect cilia, only cells expressing very low levels of GFP-CETN1-DIAPHs were selected, where the GFP signal was restricted to the basal body (cells expressing higher levels had GFP signals mislocalized to other cellular compartments or the cytosol and were not counted). Cells transfected with GFP-CETN1-DIAPH2 and GFP-CETN1-DIAPH3 had cilia that were longer or had a bulbous protrusion at their distal tips (Fig. 3*B*). In cells expressing CETN1-DIAPH2, elongated cilia were longer than cilia with bulbs or control cilia, while bulbed cilia were shorter than control cilia (Fig. 3*C*). For cells expressing CETN1-DIAPH3, bulbed cilia were the same length as control cilia, while cilia without bulbs were longer than controls (Fig. 3*C*). As further evidence that this was not related to the centrin tag, we targeted DIAPH3 with a centrosome-targeting PACT domain in hTERT-RPE cells and saw a similar increase in bulbed and elongated cilia (Fig. 3, *D*–*F*). As with centrin as the targeting vector, nonbulbed cilia were longer than bulbed cilia or controlled cilia (Fig. 3, *E* and *F*).

We focused our subsequent studies on DIAPH3, since DIAPH3 had been shown to regulate endosome trafficking (53, 66). Also, previous studies showed DIAPH3 overexpression caused a hearing defect due in part to the remodeling of the microtubule meshwork of the inner hair cell, which might be due to a kinocilium defect (56, 57, 58). In addition, the residues responsible for actin and microtubule-specific functions of DIAPH3 had been determined, and we could therefore characterize their relative contributions (51). To ensure that the expression of transgenes was comparable, we examined the level of GFP-CENT1 versus GFP-CENT1-DIAPH3 at the ciliary base (Fig. S3, *A* and *B*). Since IFT-based transport of tubulin regulates elongation of the axoneme during ciliary growth (13), we examined IFT88 intensity as a proxy for anterograde trafficking and found an increase of IFT88 intensity in the cilia of cells transfected with CETN1-DIAPH3 (Fig. S3, *C* and *E*) in comparison to the control cells transfected with CETN1-GFP. In addition, there was an increase in the intensity of PC2 in cilia of cells with basal body targeted DIAPH3, suggesting an increase in trafficking of PC2 to cilia (Fig. S3, *D* and *E*). As well, there was an increase in acetylated tubulin in the cilia of cells transfected with CETN1-GFP-DIAPH3 (Fig. S3*E*). Interestingly, the bulbous tips of cilia showed an increase of acetylated tubulin (Fig. S3*F*), indicating that bulbs comprise a large amount of acetylated tubulin. Using intensity line scans, the intensity of acetylated tubulin in bulbed cilia or nonbulbed cilia was examined (Fig. S3*G*). Overall, an increase in acetylated tubulin levels in elongated and bulbous cilia was seen upon targeting DIAPH3 proteins to the basal body in comparison to control GFP-CETN1 (Fig. S3, *E–G*). Altogether, it appears that DIAPH2 and DIAPH3 proteins control cilia length and morphology in part by regulating transport within cilia.

### DIAPH3 is needed for trafficking cargos to the base of cilia

**Figure 4:**
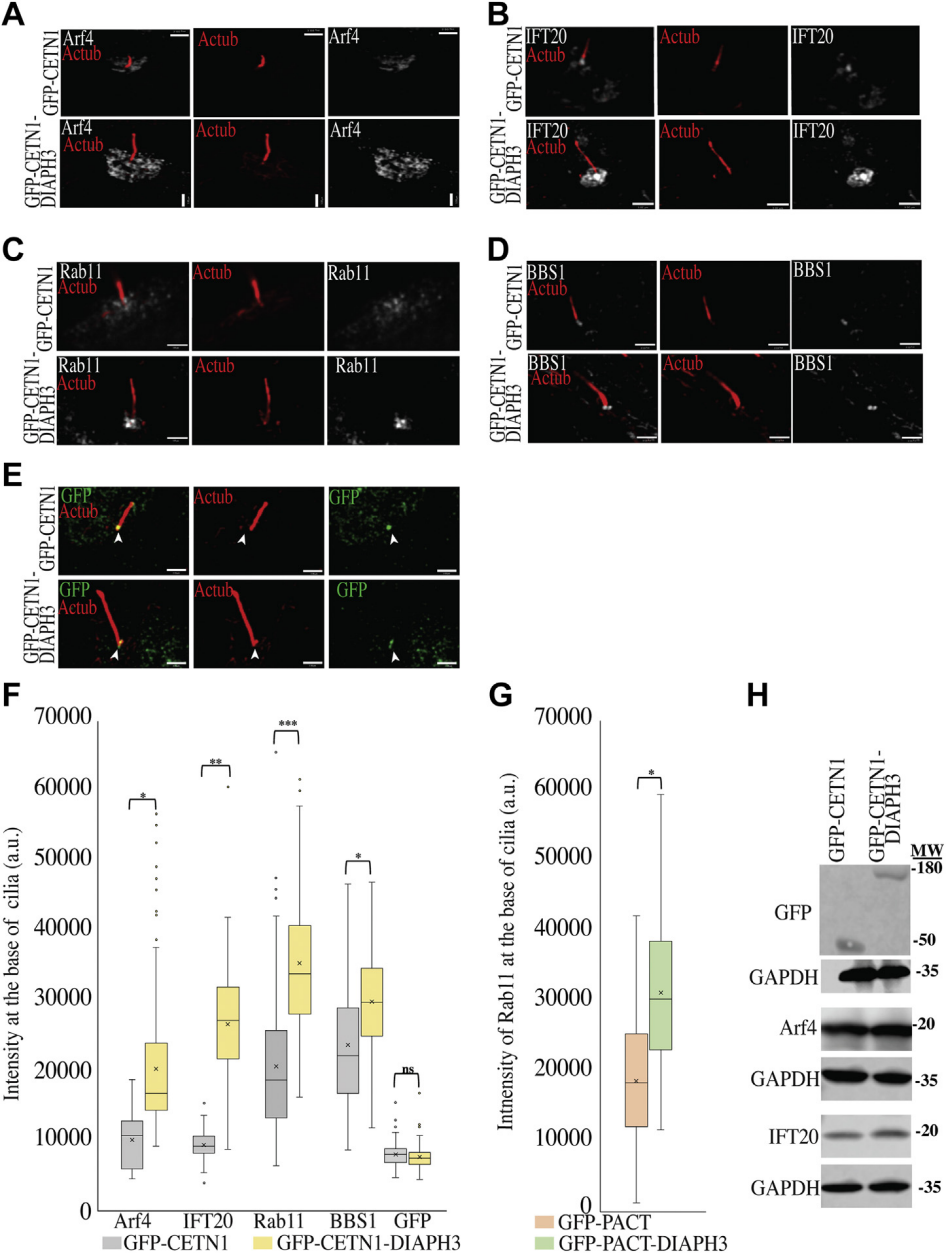
**Trafficking of post-Golgi and endosomal vesicles is increased upon targeting DIAPH3 to the base of cilia.***A–G*, targeting DIAPH3 to the base of cilia increased cargo trafficking. hTERT-RPE1 cells were transfected with GFP-CNTN1 or GFP-CNTN1-DIAPH3, and after 24 h of serum starvation, cells were fixed and stained for acetylated tubulin, GFP and (*A*) Arf4, (*B*) IFT20, (*C*) Rab11, (*D*) BBS1, or (*E*) GFP. Scale bars are 3 μm for each image. *F*, quantifications of the total protein fluorescence intensity scan at the base of cilia was conducted for Arf4, IFT20, Rab11, BBS1, or GFP. *G*, DIAPH3 targeted to the ciliary base *via* PACT increases the recycling endosome (Rab11) protein level at the base of the cilia. Cells were transfected with GFP-PACT or GFP-PACT-DIAPH3 and after 24-h serum starvation, were fixed and stained for acetylated tubulin, GFP, and Rab11. Quantification of the Rab11 fluorescence protein intensity scan was conducted. For *panels F* and *G*, box and whisker plots (as defined in Fig. 1*C*) are shown. *H*, targeting DIAPH3 to the base of cilia has no effect on protein levels of Arf4 or IFT20. Cells were transfected with GFP-CNTN1 (control) or GFP-CNTN1-DIAPH3, and samples were western blotted for GFP, GAPDH, Arf4, and IF20. Three independent experiments were conducted with n = 50 cells each, ∗*p* < 0.05, ∗∗*p* < 0.01, ∗∗∗*p* < 0.005, ∗∗∗∗*p* < 0.001. Two-tailed *t*-test analysis was done to compare GFP-CNTN1 control with GFP-CNTN1-DIAPH3.

We previously showed that DIAPH1 affected both trafficking to the cilium and also within it, and here we have shown that DIAPH3 regulates trafficking of cargo within cilia. We therefore examined whether DIAPH3 proteins may also be involved in trafficking cargo to cilia. DIAPH3 has been shown to interact with endosomes, and actin dynamics controlled by RhoB and DIAPH3 can govern vesicle trafficking (53, 66), raising the possibility that a function of DIAPH3 in endosome trafficking may be critical for trafficking cargo to primary cilia. Therefore, we examined whether DIAPH3 targeted to the basal body would affect trafficking of vesicles to the ciliary base. Key players in mediating trafficking of post-Golgi vesicles to the ciliary base are IFT20 and Arf4 proteins, and both were found to be increased upon targeting DIAPH3 to the base (Fig. 4, *A*, *B* and *F*) despite comparable expression levels of GFP-CETN1 and GFP-CETN1-DIAPH3 (Fig. 4, *E* and *F*). Additionally, we observed an increase in the level of a protein marker that is associated with recycling endosomes (Rab11) following targeting of DIAPH3 (Fig. 4, *C* and *F*). Similar results were seen by targeting DIAPH3 to the ciliary base using a PACT tag, indicating that this effect is not related to the targeting mechanism (Fig. 4*G*). The increased accumulation was not caused by changes in expression levels since western blots showed no change in Arf4 or IF20 protein levels upon targeting DIAPH3 to the base of cilia; thus, the increase in Arf4 and IFT20 at the base of cilia is due to increased trafficking (Fig. 4*H*). Since the BBS proteins localize to the ciliary base and work in trafficking cargo in IFT-mediated transport into (67), and out of, primary cilia (38), we examined the level of BBS in cells with basal body targeted DIAPH3 and observed an increase in the level of BBS1 of the octameric BBSome (Fig. 4, *D* and *E*). Trafficking of cargos to the cilium is regulated by microtubule and actin networks around the cilium (68), so we examined the level of actin and microtubules and detected an increase in the total level of α-tubulin and acetylated tubulin in the vicinity of the base (Fig. S4, *A–C*). DIAPH3 has been shown to preferentially nucleate β-actin filaments (69), but we detected no change in the intensity of γ-actin or β-actin in the presence of DIAPH3 (Fig. S4, *E* and *F*). Collectively, these data indicate that DIAPH3 proteins localized at the base of cilia influence the recruitment of post-Golgi and endosomal vesicles and stabilize microtubules in their vicinity.

### Microtubule-dependent functions of DIAPH3 are required for cilia maintenance

**Figure 5:**
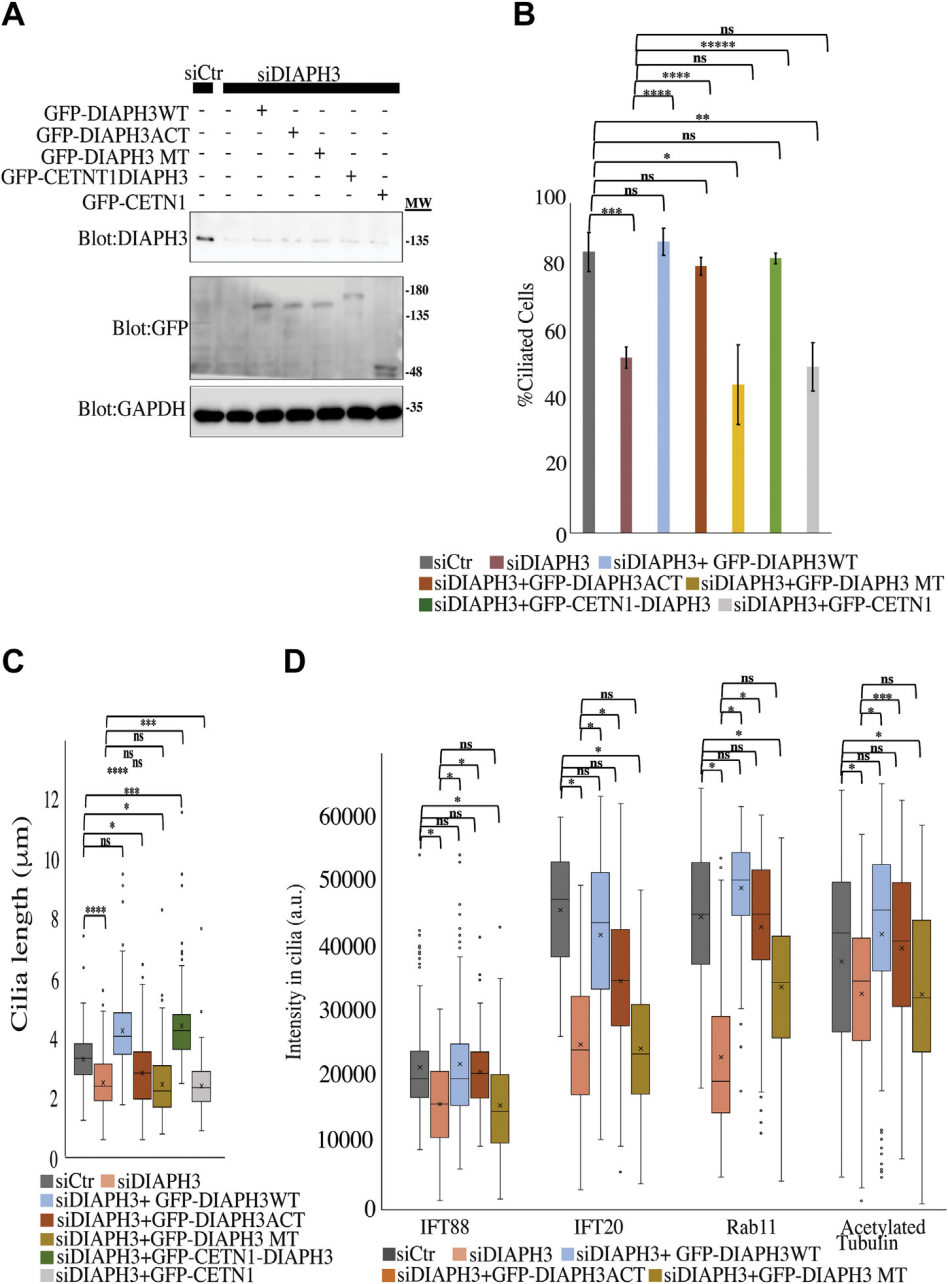
**Microtubule function of DIAPH3 regulates ciliogenesis and cilia length.***A–C*, disruption of microtubule but not actin functions of DIAPH3 negatively impacts ciliogenesis and cilia length. hTERT-RPE1 cells were transfected with siCtr or siDIAPH3, and cells were rescued using wild-type constructs of GFP-DIAPH3WT (wildtype), GFP-DIAPH3ACT (mutation preventing actin polymerization), GFP-DIAPH3MT (mutation preventing microtubule stabilization), GFP-CETN1-DIAPH3, or GFP-CETN1. *A*, western blots of samples were blotted for human-specific anti-DIAPH3, GFP, and GAPDH. Quantifications for (*B*) ciliation and (*C*) cilia length were conducted. *D*, reduction of IFT20, Rab11, and IFT88 levels at the cilia following DIAPH3 depletion cannot be rescued by DIAPH3MT. Cells were starved for 24 h, fixed, and stained for acetylated tubulin, GFP, and IFT20 or Rab11 or IFT88. For *panels C* and *D*, box and whisker plots (as defined in Fig. 1*C*) are shown. Error bars represent ± SD of three independent experiments; n = 50 each, ∗*p* < 0.05, ∗∗*p* < 0.01, ∗∗∗*p* < 0.005, ∗∗∗∗*p* < 0.001. Two-tailed *t*-test analysis was done to compare siCtr control with all samples or to compare siDIAPH3 with all samples. For *panel C*, one-way ANOVA statistical testing was also conducted on the all samples excluding the siCtr (*p* = 0.0001) or including siCtr (*p* = 0.000004) indicating that a significant difference was detected among populations.

Since DIAPH3 proteins regulate both actin polymerization and microtubule stabilization, we assessed the relative contributions of each in regulating ciliogenesis and cilia length. To do so we designed mutant forms of DIAPH3 unable to promote actin polymerization (I704A) or unable to stabilize microtubules DIAPH3 (Y713G, E714G, K715G, A717G) (51) and named them DIAPH3ACT and DIAPH3TM, respectively. hTERT-RPE1 cells were depleted of DIAPH3 and rescued with either GFP-tagged wild-type (DIAPH3WT), DIAPH3ACT or DIAPH3MT. In addition, we attempted to rescue depletion with CETN1-DIAPH3WT or with CETN1 alone (Fig. 5). DIAPH3WT, DIAPH3ACT, and CETN1-DIAPH3 were able to rescue ciliogenesis frequency defects caused by DIAPH3 depletion, but DIAPH3MT and CETN1 were not (Fig. 5, *A* and *B*). DIAPH3WT and CETN1-DIAPH3 were also able to rescue the reduction of cilia length, while neither of the mutant constructs was capable (Fig. 5*C*). When we examined specific trafficking effects, the results were more complex. DIAPH3MT was unable to rescue decreased trafficking of IFT20, IFT88, or Rab11 in cell depleted with DIAPH3 (Fig. 5*D*). Hence, ciliogenesis depends on microtubule-based functions while length is dependent on both actin and microtubule roles.

## Discussion

Recently, we showed that DIAPH1, member of DIAPH formin group, regulates ciliogenesis and cilia maintenance in an actin polymerization and microtubule stability-dependent manner (44). Given the similarity between DIAPHs 1, 2, and 3, and since mutations of DIAPH2 and DIAPH3 in humans give phenotypes commonly seen in ciliopathies, we reasoned that DIAPH2 and DIAPH3 proteins may also play roles in ciliogenesis and cilia maintenance. Our data indicate that, like DIAPH1, DIAPH2 and DIAPH3 localize at the base of the cilia. Moreover, they appear to regulate cilia maintenance by mediating trafficking of post-Golgi and recycling endosomal vesicles, which have previously been shown to be important in ciliogenesis and maintenance (27).

Previous work showed that the formins DAAM1 and INF1 were also linked to motile and primary cilia respectively (42, 43). As well, the microtubule end binding protein EB family that regulates microtubule stability in conjunction with DIAPH proteins has been implicated in ciliogenesis and localized to the base of cilia (70, 71). EB proteins play roles in microtubule minus-end anchoring at the basal body and in turn facilitate vesicular trafficking to the base of cilia (71). The involvement of DIAPH2 and DIAPH3 in trafficking vesicles to the ciliary base was shown by decreases in the accumulation of IFT20 and Rab11 levels in cilia following their depletion and complementary increases in their accumulation when DIAPHs were targeted to the base of cilia with CETN1 or PACT targeting sequences. Therefore, we speculate that DIAPH2 and DIAPH3 contribute to microtubule minus-end anchoring or microtubule growth, creating tracks for vesicle trafficking to the basal body. Indeed, targeting of DIAPH3 to the basal body increased the accumulation of microtubules at cilia. Furthermore, expression of a mutant DIAPH3 defective for microtubule stabilization decreased the level of IFT20 and Rab11 at the base of cilia when compared with the wildtype DIAPH3, indicating the importance of DIAPH3’s function in stabilizing the microtubule network for vesicle traffic to cilia. Further studies will be needed to define the mechanisms by which DIAPH3 recruits vesicles to base of cilia and how this might play role in cilia growth.

Targeted expression of DIAPH3 at the base of cilia, as was seen for DIAPH1 (44), results in an increase in cilia trafficking proteins and the elongation of cilia and in some cases, the formation of bulbous tips. The correlation of increased transport proteins and increased length is consistent with the supply-limitation model of cilia growth, where the availability of cargoes regulates IFT cargo loading and cilia length (72) and with previous studies linking DIAPH3 to vesicle trafficking (53). Bulb formation may occur if membrane addition exceeds tubulin polymerization, resulting in membrane blebbing. It is formally possible that bulged cilia are due to curling of microtubules within the tips, although this seems unlikely given the rigidity of microtubules and their susceptibility to breakage under mechanical stress caused by bending. However, it is formally possible since acetylation of microtubules increases lattice plasticity to make them more flexible and less susceptible to breakage (73, 74).

Previous studies have shown that loss of kinocilia, primary cilia-like structures on hair cells, results in misformed and misoriented stereociliary bundles as well as mispositioned basal bodies and hearing loss (75, 76). For example, loss of primary cilia-related proteins *Ift88* and *kif3* impact stereocilia bundle orientation (76, 77). Interestingly, overexpression of wild-type DIAPH3 in mice also results in defects in stereociliary bundles due to abnormal distribution of microtubules and misformed or misoriented kinocilia (56, 57, 58). As we have shown, DIAPH3 function in microtubule stabilization is important for the maintenance of cilia by regulating post-Golgi and recycling endosome trafficking to the base of cilia. Hence, it is possible that the human mutations in DIAPH3 resulting in DIAPH3 overexpression and hearing loss might be due in part to the disruption of DIAPH3 function at the kinocilium.

DIAPH2 also showed localization at the base of cilia and appeared to be involved in ciliogenesis and cilia maintenance. It is remarkable that each of the three DIAPH proteins appears to be required in a nonredundant manner for the same phenomena, as depletion of each of them results in similar phenotypes, yet depletion of all of them is not additive, and their individual depletion can only be rescued by the specific isoform. This suggests that all three proteins act independently, but within a similar process. Since ciliogenesis consists of multiple steps that include transport, docking, and fusion of both post-Golgi and endosomal vesicles (14, 16, 78), each DIAPH isoform could have a critical function at a different step of ciliogenesis. Interestingly, when we tracked the intensity of different DIAPHs with the basal body marker centrin at different times after inducing ciliogenesis, we found that the level of each changed in distinct ways. In addition, DIAPH2 was recently shown to contain two microtubule-binding domains that appear to control microtubule dynamics by a different mechanism (79) than DIAPH1 or DIAPH3, supporting the idea that it could function differently in ciliogenesis. This nonredundancy of DIAPHs is consistent with previous studies showing that DIAPH1–3 play nonredundant roles in the capture of cortical microtubules and in directed cell migration (80). More work is needed to distinguish the specific roles of each DIAPH in cilia growth and maintenance.

## Experimental procedures

### Cell culture and transfections

Immortalized human retinal pigmental epithelial cells (hTERT-RPE1) and human foreskin fibroblast (HHF1) cells were obtained from American Tissue Culture Collection (Manassas, Virginia, United States). hTERT-RPE1 cells were cultured in DMEM/F12 with 10% fetal bovine serum (FBS), while HFF1 cells were cultured in in DMEM with 15% FBS. All cells types were grown at 37 °C with 5% CO2. To initiate ciliogenesis, cells were washed twice in phosphate-buffered saline (PBS) and serum starved in DMEM/F12 or DMEM overnight to induce the formation of primary cilia. Cells were transfected with constructs and were incubated overnight for 18 h and serum starved for 24 h before they were examined by immunofluorescence staining, live microscopy, or immunoprecipitation. Primary nasal epithelial cells were isolated and cultured as described previously (81). For knockdown experiments, cells were electroporated with siRNA and were incubated for 48 h and later examined for efficient protein depletion. For overexpression and rescue, hTERT-RPE cells were transfected with Lipofectamine 3000 (Invitrogen, Burlington, Ontario, Canada). For drug treatment with Rho inhibitor, Rho inhibitor I (CT04, Cytoskeleton Inc, Denver, Colorado, United States) was used. hTERT-RPE cells were serum starved for 24 h and then treated for 2 h or 4 h with 1.0 μg/ml of Rho inhibitor I, which causes ADP ribosylation of Rho Asn-41.

### Plasmid and siRNA

The centrin or PACT-tagged DIAPH3 constructs were designed with similar methods as previously described (44). Myc-tagged DIAPH1 (mDia1) and DIAPH3 (mDia2) were generous gifts from John Copeland, University of Ottawa, and myc-tagged DIAPH2 (mDia3) was a generous gift from Yinghui Mao, Columbia University. siRNA#1 for human DIAPH1 (CTM-261432:PALOA-00007), DIAPH2 (CTM-261432:PALOA-00007), and DIAPH3 (J-018997–05) was obtained from Dharmacon, Inc Lafayette, Colorado, United States, while siRNA#2 for human DIAPH2 (DIAPH2-homo-2946 A) and DIAPH3 (DIAPH3-homo-1368 A) was obtained from GenePharma Shanghai, China. All siRNAs were screened for their ability to knockdown protein by western blotting, and the most efficient siRNA was used for subsequent experiments including western blots.

### Western blotting

Immunoblotting procedures were conducted as previously described (44), and membranes were blocked with 5% nonfat milk in Tris-buffered saline with 0.05% Tween 20 (TBST) and incubated with primary and then secondary antibodies for 1 h each. The dilutions of rabbit antibodies for western blots were as follows: DIAPH1 at 1:500 (Abcam, Cambridge, Massachusetts, United States; ab133683), DIAPH2 at 1:1000 (Abcam, Cambridge, Massachusetts, United States; ab102841), DIAPH3 at 1:500 (Proteintech, Rosemont, Illinois, United States; 13342-1-AP); RB (phospho S807) at 1:20,000 (Abcam, Cambridge, Massachusetts, United States; ab184796); and GFP at 1:1000 (Invitrogen, Burlington, Ontario, Canada; A-11122). The dilutions for mouse antibodies were as follows: Myc at 1:1000 (Covance, Toronto, Ontario, Canada; 9E10), GAPDH at 1:50,000 (Millipore, Oakville, Ontario, Canada; AB2302). The secondary antibodies, which were all horseradish peroxidase (HRP)-conjugated goat antibodies, were used at dilution of 1:5000 to detect either rabbit or mouse primary antibodies (Jackson ImmunoResearch Laboratories, Inc, West Grove, Pennsylvania, United States).

### Immunofluorescence

Cells grown on coverslips were fixed with either paraformaldehyde (PFA) or ice-cold methanol depending on the antibody. For the PFA fixation, cells were fixed with 1% PFA in 1XPBS or 3% PHEM buffer (60 mM Pipes-KOH, pH 6.9, 25 mM Hepes, 10 mM EDTA, and 2 mM MgCl_2_, 0.1% Triton X-100) for 20 min at room temperature followed by PFA inactivation and permeabilization buffer C (PBS with 25 mM glycine, 25 mM ammonium chloride, and 0.1% Triton X-100) for 10 min. For methanol fixation, cells were fixed with ice-cold methanol for 3 min. Cells from either fixation condition were then blocked in PBS with 5% FBS or horse serum for 20–30 min. Cells were then incubated with primary antibodies for 1 h, washed twice with 1XPBS, and then an appropriate secondary antibody was added for 1 h at room temperature. Primary antibodies for DIAPH1 (ab11173), DIAPH2 (ab1032841), Polycystin2 (PC2) (ab214317), Polycystin1 (ab235963 or ab74115), GFP (ab13970), Arf4 (ab171746), EB3 (ab53360), and EB1 (ab53358) were obtained from Abcam, Cambridge, Massachusetts, United States. BBS1 (21118-1-AP), Arl13B (1711-1-AP), DIAPH3 (14342-1-AP), IFT20 (13615-1-AP), Rab11 (20220-1-AP), and IFT88 (139671-1-AP) were obtained from Proteintech (Rosemont, Illinois, United States). Myc (9E10) was from Covance (Toronto, Ontario, Canada). Antibody against basal body Centrin1 (20H5; 04–1624) was from EMD Millipore and acetylated tubulin (T7451) from Sigma-Aldrich (St Louis, Missouri, United States). All fluorescently conjugated goat secondary antibodies (AlexaFluor 488, 564 and 684 dyes) from Invitrogen were used at 1:500. After secondary labeling, all coverslips were washed three times with PBS. Cells were counter-stained with Hoescht (Sigma-Aldrich, St Louis, Missouri, United States) to label DNA. Finally, coverslips were mounted using fluorescence mounting medium from Dako (Glostrup, Denmark).

### Microscopy

Quorum spinning disk confocal was used to image cells. The microscope is equipped with EMCCD digital camera (Hamamatsu ImagEM). Images were acquired using 63X oil immersion objective (HCX Plan Apochromat with numerical aperture of 1.40–0.7; Leica), and the equipment was driven by Volocity acquisition software (Quorum Technologies, Puslinch, Ontario, Canada) and powered by a Power Mac G5 (Apple).

### Quantification

Volocity software was used to measure cilia length and region of interest intensity. Using the line scanning tool, the length of cilia stained with acetylated tubulin was measured, while fluorescence intensity of specific regions of interest was determined using the ROI tool. The background was subtracted from all fluorescence intensity scans. For each experiment, 50 or more cilia or cells were counted. All data were plotted, and *p*-values were calculated using Excel.

For all figures with intensity measurements, the relative ciliary intensities were averaged over the entire length of the cilium regardless of length. The ROI scan tools in Volocity software were used to measure average intensity of ciliary proteins within a specific region and in this case that included the entire cilium. Please see cartoon illustration in Fig. S3*E*. Hence, cilium length is normalized by capturing and averaging the intensity of the entire cilium. In cases where we measure intensity at the base or tip of cilia, these regions of interest are not normalized for length. The line intensity scan on Fig. S3*G* compares intensity of acetylated tubulin along the length of cilia (from base to tip of cilia). Cells expressing the fusion protein GFP-CENT1 or GFP-CENT1-DIAPH3 were immunostained for GFP, and the level of GFP signal at the base of cilia was measured to ensure comparable expression. If cells overexpressed the GFP-CENT1 fusion constructs such that they were observed outside the basal body, those cells were excluded from counting.

## Data availability

All data are contained within the article.

## Supporting information

This article contains supporting information.

## Conflict of interest

The authors declare that they have no conflicts of interest with the contents of this article.
