# Supplementary material for: Nonredundant roles of DIAPHs in primary ciliogenesis
Source: J Biol Chem. 2021 Apr 17;296:100680. doi: 10.1016/j.jbc.2021.100680 (PMC8122175; doi:10.1016/j.jbc.2021.100680)
Supplement: Figures S1–S4 [file mmc1.doc]

**Supplementary Data**

**Non-redundant roles of DIAPHs in primary ciliogenesis**

Oliva Palander^1,3^, Adam Lam^1^, Richard F. Collins^1^, Theo J. Moraes^2,4^, and William S. Trimble^1,3*^

Programs in ^1^Cell Biology and ^2^Translational Medicine, Hospital for Sick Children, Toronto, Ontario, Canada, M5G 1X8

Departments of ^3^Biochemistry and ^4^Paediatrics, University of Toronto, M5G 1A8

^*^Corresponding author: William S. Trimble

E-mail: [wtrimble@sickkids.ca](mailto:wtrimble@sickkids.ca)

**Running title**: DIAPHs regulate ciliogenesis

**Keywords:** cilia, DIAPH3, DIAPH2, ciliogenesis, cilia maintenance.

Figure S1


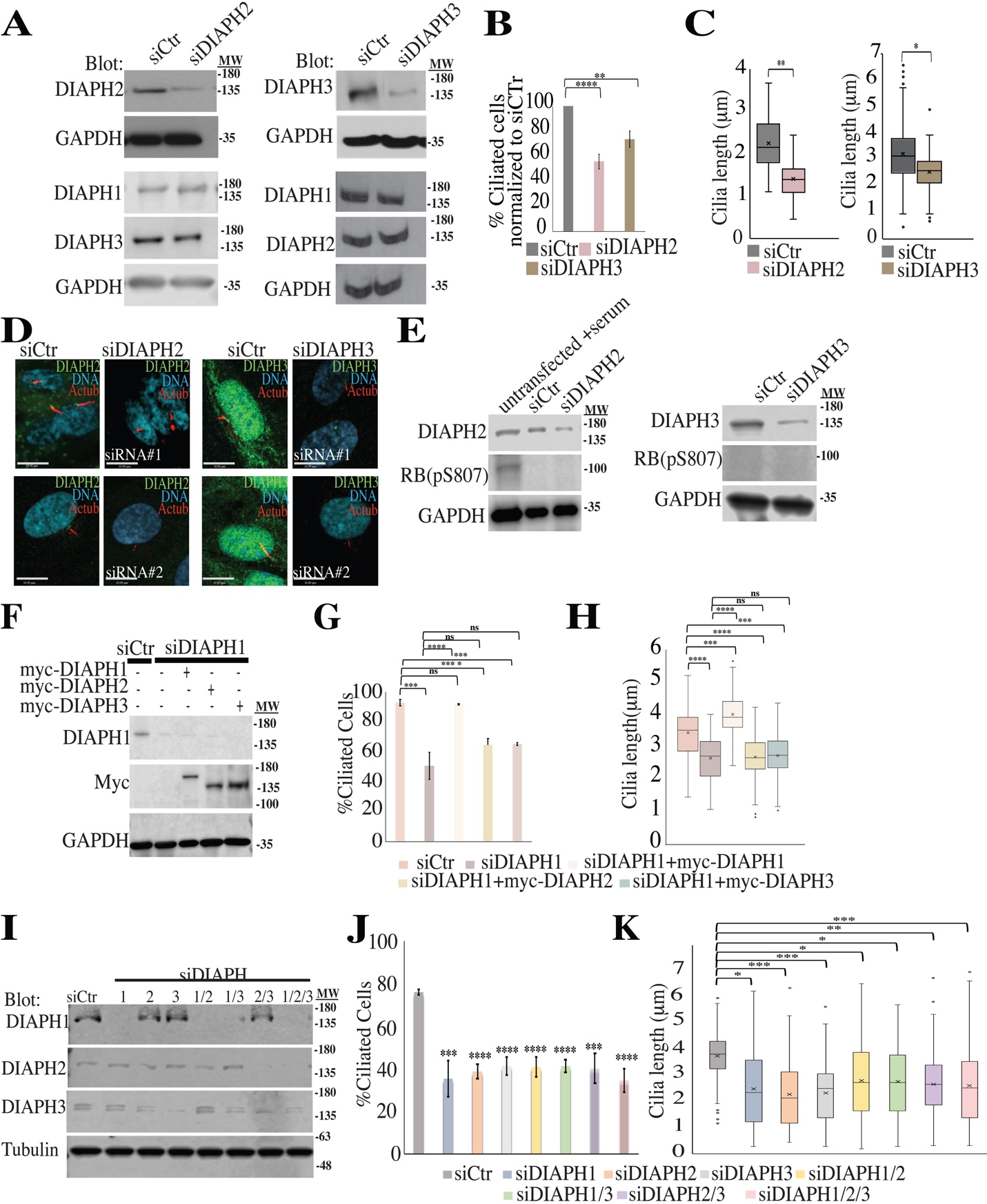


Figure S1: DIAPH2 and DIAPH3 function in ciliogenesis

(**A-C**) Depletion of DIAPH2 and DIAPH3 impairs ciliation and cilia length. hTERT-RPE1 cells were transfected for 48 hours with scrambled control (siCtr) or siRNA #2 targeted toward human DIAPH2 (siDIAPH2#2) or human DIAPH3 (siDIAPH3#2). Cells were serum starved for 24 hour. (**A**) Western blots of DIAPH2 or DIAPH3 depleted lysates were probed for DIAPH1, DIAPH2 or DIAPH3, and GAPDH as a loading control. (**B**) Quantification of the percent of cells with cilia. (**C**) Box-and-whisker plot for the quantification of cilia length (as defined in Figure 1C). The lengths of cilia from ciliated cells were measured in each condition using a line scan tool by measuring the length of the acetylated tubulin signal. (**D**) Loss of DIAPH2 and DIAPH3 at the base of cilia upon siRNA treatment. hTERT-RPE1 cells were transfected for 48 hours with siCtr or siRNA targeted towards two different human DIAPH2 or DIAPH3, and serum starved for 24 hours. siRNA#1 and siRNA#2 for siDIAPH2 and siDIAPH3 were purchased from Dharmacon and GenePharma, respectively. Cells with control, DIAPH2 or DIAPH3 siRNA treatment were fixed and stained for DIAPH2 and Actub. Scale bars are 10 μm for images. (**E**) No change in phosphorylation level of Rb upon depletion of DIAPH2 or DIAPH3. hTERT-RPE1 cells were either un-transfected or transfected for 48 hours with scrambled control (siCtr) or siDIAPH2 or human siDIAPH3. All samples were serum starved for 24 hours with exception for untransfected sample. Western blots of cell lysates for untransfected, siCtr, siDIAPH2 or siDIAPH3 were probed for Rb phosphorylated S807 protein labeled as Rb(pS807), DIAPH2 or DIAPH3, and GAPDH as a loading control. (**F-H**) Impairment of ciliation and cilia length by DIAPH1 depletion could not be rescued by other DIAPH isoforms. hTERT-RPE1 cells were transfected with scrambled control (siCtr) or siRNA targeted toward human DIAPH1 (siDIAPH1) and cells were rescued by expressing wildtype murine orthologs of DIAPH1, DIAPH2 or DIAPH3, each tagged with myc. (**F**) Western blots of rescued cells blotted for DIAPH1, myc and GAPDH (note that human-specific anti-DIAPH1 does not cross-react with transfected murine ortholog). (**G**, **H**) Quantification of ciliation and cilia length in hTERT-RPE cells treated with siRNA for 48 hours. (**I**-**K**) Single or co-depletion of DIAPH isoforms impairs ciliation and cilia length to a similar extent. hTERT-RPE1 cells were transfected with scrambled control (siCtr) or siRNA targeted toward human DIAPH1 (siDIAPH1#1), human DIAPH2 (siDIAPH2#1), human DIAPH3 (siDIAPH3#1) or various combinations. (**I**) Western blots of depleted cells were probed for tubulin, DIAPH1, DIAPH2 or DIAPH3. (**J**, **K**) Quantification of ciliation and cilia length in hTERT-RPE1 cells treated with siRNA for 48 hours, respectively. For panels H and I, one-way ANOVA statistical testing was also conducted on all samples excluding the siCtr. P =0.76 (H) and P = 0.44 (I) indicating that no significance difference was detected among populations of single or co-depleted samples of DIAPH1-3. For all quantifications, error bars represent ±SD of three independent experiments; n=50. Two tailed t-test analysis was done to compare all samples, *p<0.05, **p<0.01, ***p<0.005, ****p<0.001.

Figure S2


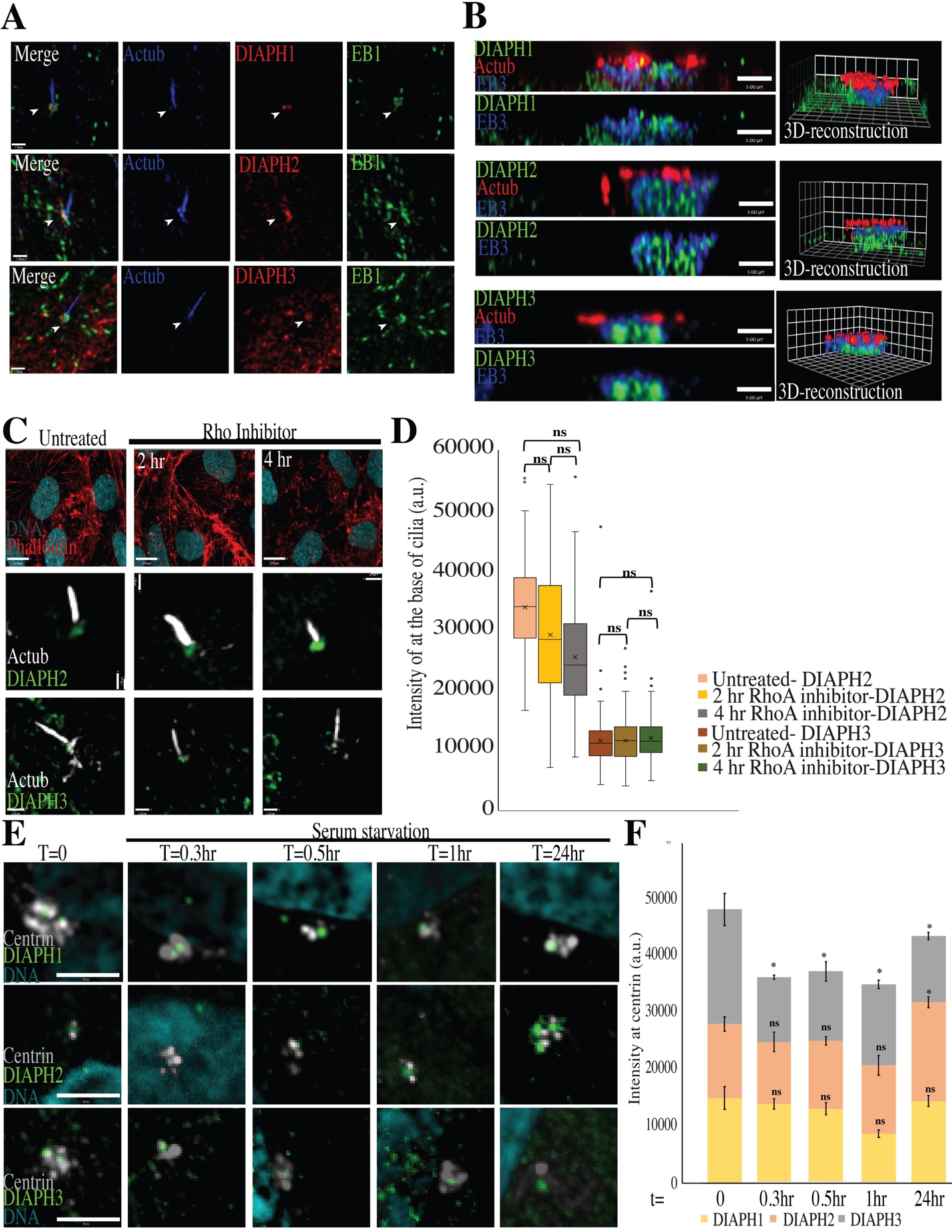


Figure S2: Localization of DIAPH isoforms at the base of primary cilia in HFF1 cells and motile cilia.

(**A**) DIAPH1-3 are localized at the ciliary base in the Human foreskin fibroblasts (hFFs). hFFs were serum starved for 24 hours, fixed and stained with antibodies to DIAPH isoforms and Actub (axonmal marker) or EB1 (basal body marker). Cells co-stained with DIAPH1(top), DIAPH2 (middle) or DIAPH3 (bottom) and Actub and EB1. Arrow heads are pointing at basal body localizations of EB1 and DIAPH1-3. Scale bars are 1.3 μm. (**B**) DIAPH1-3 are localized at the base of the motile cilia. Left of the panel D shows X-Z localization of DIAPH1-3 at the base of motile cilia in nasal epithelium cells. Motile cilia of primary human nasal epithelial cells were fixed with 4% formaldehyde in PBS and stained for DIAPH1 (top), DIAPH2 (middle), DIAPH3 (bottom) and EB3 (basal maker) and Actub (axoneme maker). Scale bars are 5μm. 3D-reconstruction image is shown at the right of panel B. (**C**-**E**) Rho signalling has no effect on DIAPH2 and DIAPH3 intensity at the base of cilia. (**C**) hTERT-REPE1 cells were serum starved for 24 hours and treated with 1.0 µg/ml Rho inhibitor I for 2hrs or 4 hrs, and stained with phalloidin and antibodies to Actub, DIAPH2 or DIAPH3. (**D, E**) Average fluorescence intensity was measured to determine relative protein levels of DIAPH2 or DIAPH3 at the base of cilia. (**F**, **G**) Time course of DIAPH recruitment during ciliogenesis for DIAPH1-3. (**F**) hTERT-RPE1 cells were serum starved for various time points (0.3hr, 0.5hr, 1hr, and 24 hrs), and cells were stained for centrin and DIAPH1, DIAPH2 or DIAPH3. Centrin was used a marker to track ciliogenesis at various time points. (**G**) Average fluorescence intensity was measured to determine the relative protein levels of DIAPH1, DIAPH2 or DIAPH3 at the centrin positive region at various time points. Error bars represent ±SD of three independent experiments; n=50 or more cells each. Two tailed t-test analysis was done to compare untreated to Rho inhibitor treated samples or T=0 to time points, *p<0.05, **p<0.01, ***p<0.005, ****p<0.001.

Figure S3


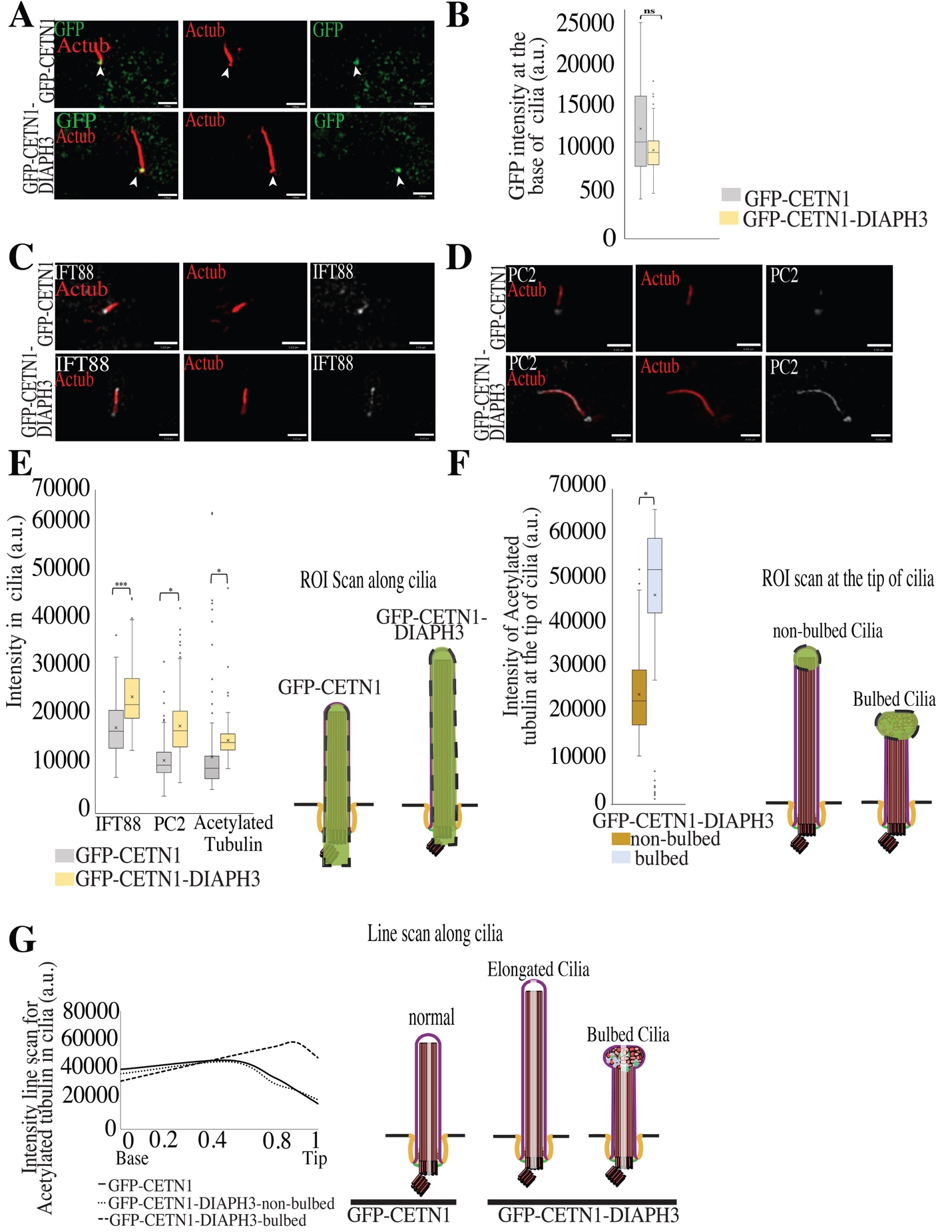


Figure S3: Basal body-targeted DIAPH3 changes trafficking and acetylated tubulin levels within cilia.

**(A**-**B)** Expression level of GFP-CETN1 and GFP-CETN1-DIAPH3 at the base of cilia. hTERT-RPE1 cells were transfected with either GFP-CETN1 or GFP1-CETN1-DIAPH3 constructs and cells were serum starved for 24 hours. (**A**) Cells are fixed and stained for GFP and acetylated tubulin. Scale bars are 3 μm for each image (**B**) Average intensity of GFP at the base of cilia was quantified and presented in box-and whisker plot. **(C-F**) Examination of ciliary components upon targeting DIAPH3 to the base of the cilia. hTERT RPE1 were transfected with GFP-CNTN1 or GFP-CNTN1-DIAPH3 and serum starved. (**C**) Cells were immunostained for acetylated tubulin, GFP (not shown) and IFT88. Scale bars are 3 μm for each image. (**D**) Cells were immunostained for acetylated tubulin, GFP (not shown) and PC2. Scale bars are 3 μm for each image. (**E**) Average intensity of IFT88, PC2 and acetylated tubulin in cilia of cells expressing GFP-CNTN1-DIAPH3 or GFP-CNTN1 was measured. ROI for the measurement of ciliary protein is shown in green. (**F, G**) Comparing acetylated tubulin levels in cilia upon DIAPH3 targeted to the base. (**F**) Quantification of the region of interest (ROI) intensity of acetylated tubulin in cilia at the tip of bulbed vs non-bulbed cilia was measured. ROI for the measurement of the ciliary protein is shown in green. (**G**) Line intensity scans of acetylated tubulin for bulbed, non-bulbed or control cilia. The cartoon illustrations show the measurement of the line intensity scan along the cilia in white. Error bars represent ±SD of three independent experiments; n=50 cells each, *p<0.05, **p<0.01, ***p<0.005, ****p<0.001. Two tailed t-test analysis was done to compare CNTN1-GFP control to other samples.

Figure S4


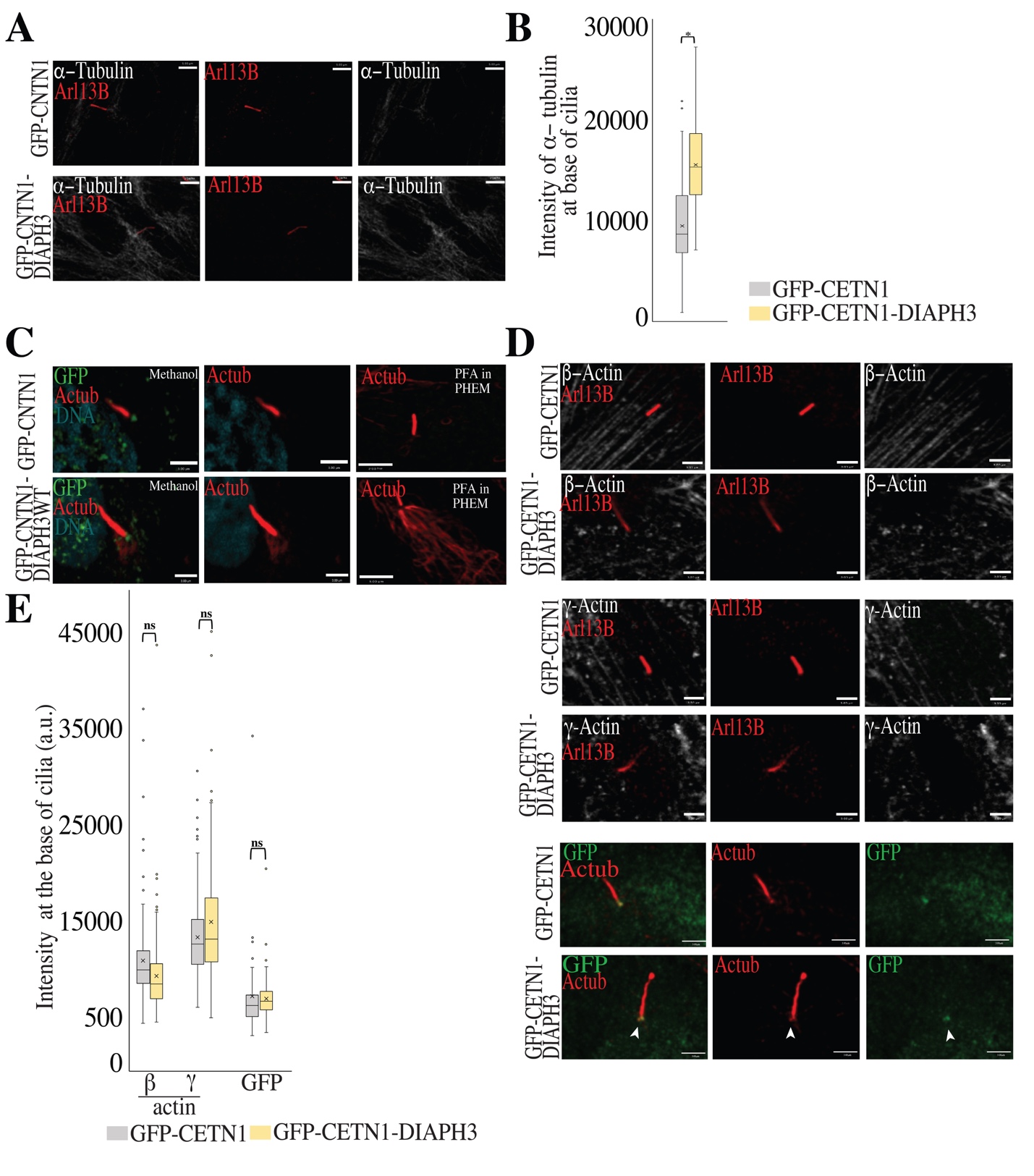


Figure S4: DIAPH3 regulates the microtubule network at the base of cilia.

(**A-C**) Increases in levels of α-tubulin and acetylated tubulin at the ciliary base upon targeting DIAPH3 to the basal body. (**A**) hTERT-RPE1 transfected with GFP-CNTN1-GFP or GFP-CNTN1-DIAPH3, and after 24 hours of serum starvation, fixation and staining for (**A**) Arl13B and α-tubulin or (**B**) acetylated tubulin and GFP. Scale bars are 3 μm for each image. (**B**) Quantification of tubulin at the cilia base was conducted for total protein intensity of α-tubulin. (**C**) Enhancement of acetylated tubulin levels at the cilia base upon targeting DIAPH3 to the base of cilia. Cells were transfected with GFP-CNTN1 or GFP-CNTN1-DIAPH3 and then fixed either with methanol or with PFA in PHEM buffer and stained for GFP and acetylated tubulin. Scale bars are 3 μm (for methanol fixation, left and middle panels) and 5 μm (PFA in PHEM fixation, right panels) for each image. (**D and E**) No change is detected in actin level or pattern upon targeting DIAPH3 to the ciliary base. hTERT-RPE1 cells were transfected with GFP-CNTN1­­ or GFP-CNTN1-DIAPH3. After 24 hours of serum starvation, cells were fixed and immunostained for acetylated tubulin, GFP, β-actin, γ-actin or Arl13B. Scale bars are 3 μm for each image. (**E**) Quantifications of γ-actin, β-actin or GFP in the vicinity of the cilia base is shown. Error bars represent ±SD of three independent experiments; n=50 each, *p<0.05, **p<0.01, ***p<0.005, ****p<0.001. Two tailed t-test analysis was done to compare GFP-CNTN1 control to GFP-CNTN1-DIAPH3.
